# Recognizing Cursive Typewritten Text Using Segmentation-Free System

**DOI:** 10.1155/2015/818432

**Published:** 2015-04-15

**Authors:** Mohammad S. Khorsheed

**Affiliations:** National Center for Robotics and Intelligent Systems, King Abdulaziz City for Science & Technology, P.O. Box 6086, Riyadh 11442, Saudi Arabia

## Abstract

Feature extraction plays an important role in text recognition as it aims to capture essential characteristics of the text image. Feature extraction algorithms widely range between robust and hard to extract features and noise sensitive and easy to extract features. Among those feature types are statistical features which are derived from the statistical distribution of the image pixels. This paper presents a novel method for feature extraction where simple statistical features are extracted from a one-pixel wide window that slides across the text line. The feature set is clustered in the feature space using vector quantization. The feature vector sequence is then injected to a classification engine for training and recognition purposes. The recognition system is applied to a data corpus which includes cursive Arabic text of more than 600 A4-size sheets typewritten in multiple computer-generated fonts. The system performance is compared to a previously published system from the literature with a similar engine but a different feature set.

## 1. Introduction

Optical character recognition (OCR) is amongst the branches of pattern recognition where a computer program attempts to imitate the human ability to read printed text with human accuracy, but at a higher speed [1]. A number of factors are pushing toward text recognition: the easy use of electronic media, its growth at the expense of conventional media, and the necessity of converting the data from the conventional media to the new electronic media. The latter motivates the vast range of OCR applications which includes automatic mail routing [2], machine processing of forms [3], bank cheques [4], printed newspapers [5], and signature verification [6].

Most optical character recognition methods for Latin text assume that individual characters can be isolated. Although this is applicable and successful for those languages, this assumption cannot be applied reliably to cursive script, such as Arabic, where the shape of the character is context sensitive. Feature extraction tackles the obstacle of cursiveness of Arabic in twofold: the global approach and the analytical approach. While global approach treats the word as a whole, extracts features from the unsegmented word, and then compares those features to a model [7, 8], analytical approach decomposes the word into smaller units called glyphs [9]. Glyphs may or may not correspond to characters, although previous research has confirmed the difficulties in attempting to segment Arabic words into individual characters [10].

A feature measures certain attributes of a glyph and then combines those measurements into a vector. Those measurements capture essential characteristics of the glyph which eliminate variations of the same glyph across various fonts and preserve properties between two different glyphs. Features could be structural, statistical, or global transformation. Structural features concern with topological and geometrical characteristics of a glyph [11, 12]. These include strokes and bays in all directions, intersections of lines, endpoints, positions of strokes and dots relative to the baseline, loops, and zigzags [13–15]. From one side, structural features are difficult to extract; in contrast, they are capable of tolerating variations in writing styles and noise [16]. Statistical features may compute the density distribution of the glyph pixels [17] or count the segments of ones and zeros, the length of each of those segments, and the ratio of how the image pixels are distributed between image parts [18]. They can also have the form of image intensity function, moments which may be invariant to geometric transformations such as scaling, rotation, and translation [19, 20]. Statistical features are easier to compute than structural features; however, they are very sensitive to noise and style variation. Global transformation features transform the representation of the image pixels from the current status into a more compact form. This includes using Fourier Descriptors (FDs) to represent the character's outer skeleton via a periodic function [21] transforming each word into a normalized polar image, then applying the two-dimensional Fourier transform to the polar image [22] or representing the boundary pixels of the character using Freeman code [23, 24].

After transferring the glyph image into a sequence of feature vectors, the next step is to classify this sequence into one of predefined clusters. Various classification methods and techniques have been applied in recognizing Arabic alphanumerical and text. These include Template Matching [25], Euclidean Distance [26], Neural Networks [27], Fuzzy Logic [28], Genetic Algorithms [29], and Hidden Markov Models (HMMs) [30]. HMMs are statistical models which are widely and efficiently implemented among applications such as speech processing, online character recognition [31], and offline character recognition [32]. The HMM can tolerate variations in time-varying patterns by providing explicit representation for these patterns. There are a number of packages which enable researchers to implement HMMs to their environments. Among those packages is the HMM Tool Kit (HTK) [33].

This paper presents a novel algorithm to extract a feature set from a one-pixel wide window that slides across the Arabic text line image from right to left. This feature set includes the segment length within each image column. The feature space is clustered using vector quantization (VQ) [34] in order to reduce the dimensionality of the problem from two dimensions to one dimension. This enables us to utilize one of the existing recognition engines; HTK.

## 2. The Proposed Algorithm

Activities conducted within the OCR system include acquiring the document image, preprocessing it, and then decomposing it into text line images, clustering the feature space into classes using VQ, coupling the discrete representation of the features with the corresponding ground truth to estimate the character model parameters. During recognition, an input line image is transferred into a feature vector sequence, clustered into a sequence of discrete symbols. This sequence is then injected into the recognition engine which outputs a stream of characters matching the text line.

The text line image is fed to the system as a two-dimensional binary array. Feature extraction applies a set of statistical measures to the line image which results in a sequence of two-dimensional feature vectors. Those feature vectors are computed as a function of a sliding one-pixel wide window scanning the line image from right to left. A set of simple features is extracted from pixels falling within that window. This feature set represents the Run-Length Encoding (RLE) of the pixel column [35]. RLE is a quick and simple algorithm to compress data. This algorithm is supported by various bitmap file formats such as PCX, BMP, and TIFF. For each repeating string of characters, the algorithm stores the character value and computes the frequency of that character within the string. The algorithm refers to these two figures as the run value and the run length. The efficiency of the algorithm to compress data highly depends on the nature of image under consideration.

**Figure 1:**
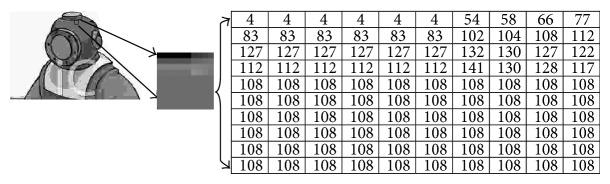
Implementing RLE to a gray image.

Figure 1 illustrates the implementation of RLE algorithm to a gray image. RLE extracts the runs of data for each segment within each column. The algorithm finds the intensity value for a pixel, the run value, and counts the number of pixels with the same value in that segment, run count. The new representation of the image using RLE may not be friendly to be utilized to train and test a recognition system. In contrast, a binary image has less pixel complexity as there are only two run values, one or zero, and therefore a more concise representation for the image. In this paper, a document image represents the binary image of one A4-size page where white is the background and black is the foreground. This document image is mostly white and hence is efficiently encoded due to the large amount of contiguous data that has the same run value.

**Figure 2:**
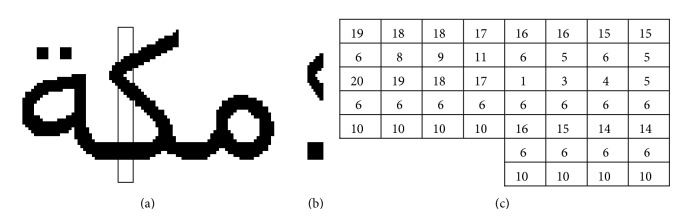
RLE implementation to binary image.

**Figure 3:**
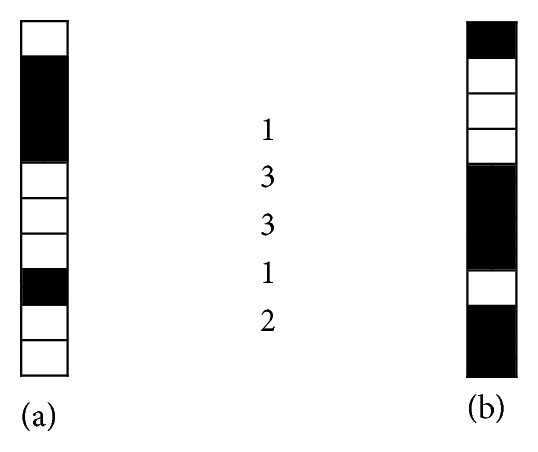
Encoding individual columns.

The document binary image is run-length encoded in a sequential process which processes the image data as a one-dimensional stream, rather than a two-dimensional map of data. This implies that the algorithm starts from the top right corner of the image, traverses the first column, and transfers each segment into a single number which represents the run count of ones or zeroes. This process iterates to all consequent columns.  Figure 2 illustrates the implementation of the proposed algorithm to a small portion of a word image.  Figure 2(b) shows a portion of a word image in Figure 2(a). Each column in the text line image is transferred into a sequence of discrete numbers where each number represents the run count of a segment of zeroes or ones as shown in Figure 2(c). There is no certain order for the segment sequence in a given column as this depends on whether the first pixel of that column is zero or one.  Figure 3 shows two columns with two different pixel combinations. The two columns have the same segment sequence and run counts. To remedy this, we assume that the first pixel in the column is zero and we count the run length accordingly. If the first pixel in the column is one then we assign zero value to the first segment. This presents consistency among all columns which is essential to clear confusion between those columns with similar segment sequences. Applying this to the columns shown in Figure 3, the first column has the same segment sequence where the second column (b) alters its segment sequence into the following: 0, 1, 3, 3, 1, 2. Though the problem of similarity is resolved now, different segment sequence sizes appear clearly here. The next section will resolve this challenge.

## 3. Implementation and Recognition Results

### 3.1. The Arabic Corpus

**Figure 4:**
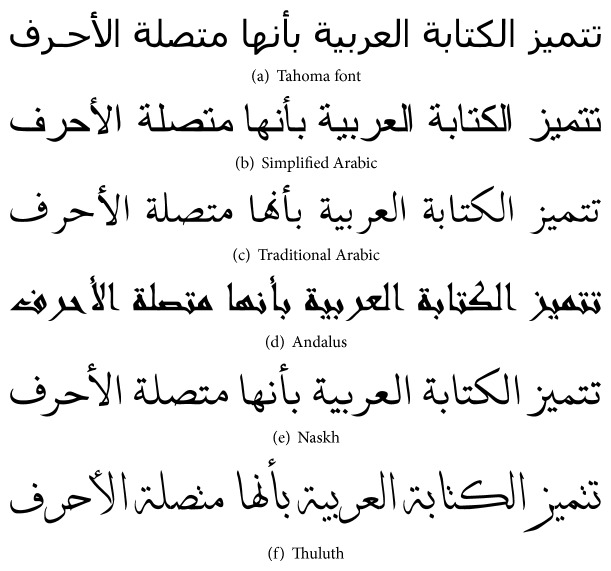
Samples from the six fonts in the corpus.

The proposed algorithm is implemented to a corpus that includes more than 600 A4-size pages of Arabic text. The content was typewritten in six different computer-generated fonts. These fonts are Tahoma, Simplified Arabic, Traditional Arabic, Andalus, Naskh, and Thuluth; see Figure 4. They cover different complexity scales ranging from Tahoma which is a simple font with no overlap or ligature to Thuluth which is very rich with challenges: overlaps, ligatures, and decorative curves. The corpus includes 15000 text line images of 116743 words and 596931 letters, not including spaces. It has line heights which are proportional to the font type and size. The line image height varies from 35 pixels to 95 pixels with different number of segments per column. Various approaches were applied to produce uniform feature vectors. Khorsheed [7] resized all line images to a single height of 60 pixels. This allows the feature extraction to produce consistent feature vectors.

**Figure 5:**
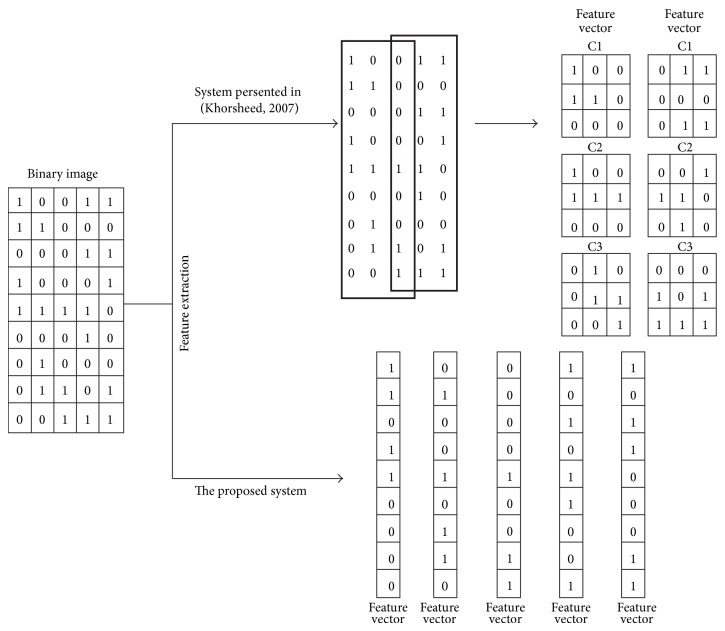
Feature extraction using the proposed algorithm and Khorsheed [7].

In this paper, we tackle this variation differently. We aim to calculate the optimal size of the feature vector or in other words the optimal number of segments per column. This is related to a number of transitions from zero (background) to one (foreground) and vice versa.  Table 1 shows numbers of transitions per column, number of columns with this transition number, and the accumulative percentage. More than 99% of the 16,287,440 columns in the corpus have six transitions at most. This means that those columns have seven runs/segments or less. Therefore, we decide to transfer each column in the line image into a seven-dimensional feature vector. Each item within that feature vector represents the run-length of the foreground or background pixels. All other transitions beyond the first six transitions from the top are discarded. The proposed algorithm produces feature vectors 3 to 5 times more than the algorithm presented in [7]. In that algorithm, the sliding window was vertically divided into cells where each cell includes 3 × 3 or 5 × 5 pixels. Three features were extracted from each cell: the intensity, the intensity of horizontal derivative, and the intensity of vertical derivative. Vertical and horizontal overlaps between cells increase the amount of features generated from an individual line image though increase the processing time.  Figure 5 illustrates the outputs of the proposed algorithm and [7] from a small portion of a text line binary image. The proposed algorithm produces 6 feature vectors, one from each column. Khorsheed [7] first slid a 3 × 3 window vertically with zero overlap which generated three cells. Those three cells were combined together to form one feature vector. The sliding window then shifted two pixels to the left which resulted in one pixel horizontal overlap. The algorithm finally produced only two feature vectors from the given binary image portion. The difference in the size of the feature vectors extracted using the two algorithms will impact training the recognition engine as we shall see next section. Both algorithms implemented VQ to map continuous density vectors to discrete simple symbols. A vector quantizer depends on a so-called codebook which defines a set of clusters each of which is represented by the mean value of all feature vectors belonging to that cluster. Each incoming feature vector is then matched with each cluster and assigned the index corresponding to the cluster which has the minimum difference value or in another words is closest.

### 3.2. The Recognition Engine

This is based on the hidden Markov model toolkit (HTK) [33]. HTK is a portable toolkit for building and manipulating hidden Markov models. Most of HTK functionality is built as C code libraries which facilitates writing scripts to execute HTK tools. The HTK tools have three phases: data preparation, training, and recognition tools. We hardcode the data preparation tools to acquire the document image, preprocess it, and then decompose it into text line images as the text line is chosen here as the unit for training and recognition purposes. The C-code also performs RLE feature extraction before converting the final result into HTK format. Data preparation tools are also responsible for mapping the output of feature extraction against predefined codebook vectors and replaced with the symbol representing the nearest codebook vector. This step transfers the text line image into a sequence of discrete symbols. It takes as input a set of feature vectors, clusters them, and uses the centroid of each cluster to define the clusters with the codebook.

The data preparation tool builds a linear structured codebook in an iterative process. Initially, there is only one cluster with a mean value of all training vectors. In each following iteration, if the total distance between the cluster members and the mean is more than a predefined threshold, the mean is then perturbed to give two means and the vectors within that cluster are rearranged according to which mean is nearest to them. This continues until the codebook size reaches the required number of clusters.

HTK recognition tool decodes the observation sequence and outputs the associated state sequence. It requires a network to describe the transition probabilities from one character model to another. Each model represents various shapes of one character in the alphabet. In this paper, we have implemented two character model schemes: monomodels and trimodels. A monomodel is context-independent where each character in the alphabet is represented by a distinct HMM. Each character in the word is separated from its preceding and succeeding neighbors. Monomodels are easy to train, as the total number of models is relatively small, and simple to label, as each label represents one character. In contrast, a trimodel is context-dependent where each model consists of a combination of three letters: the recognized letter and its preceding and succeeding neighbors in the context.

Table 2 shows the system performance of four different experiments all executed using 1024 codebook size. Two of those experiments were performed using part of the training dataset as a test dataset. This illustrates the system capability to learn, meaning apparent relationships in the training data can be identified. The other two experiments were performed using independent test dataset of the training dataset. This assesses if the relationships previously identified can be held in general.

As shown in Figure 5, the proposed algorithm produces feature vectors 3 to 5 times more than [7]. This enables fine tuning the recognition engine parameters more accurately as illustrated in Table 3. The more states a model has the more data it needs to reestimate its parameters. This is also essential for trimodels as there are around 9400 models each has its own set of parameters. Table 3 shows a huge drop in the recognition rate for [7] when the number of states per model is 16. This is due to the lack of adequate number of feature vectors to tune the recognition engine parameters. In contrast, the proposed algorithm does not suffer from this problem as shown in the same table.

Table 4 shows the system performance for each of the six fonts in the corpus using two feature extraction methods: the proposed algorithm and [7]. As illustrated, the proposed algorithm outperforms [7] at all fonts thanks to the overwhelming number of feature vectors extracted from the line images which enable the recognition engine, using the proposed algorithm, to grasp the fine variations from various fonts and writing styles.

## 4. Conclusions

This paper presented a novel approach to extract features from the text line images. The proposed algorithm is a segmentation-free and uses run-length encoding (RLE). The performance of the proposed approach was assessed using a corpus including cursive Arabic text typewritten in various computer-generated fonts and a recognition engine based on Hidden Markov Models Tool Kit (HTK). The system was capable of learning complex ligatures and overlaps. Finally, a comparison was conducted between the proposed algorithm and another algorithm which extracted intensity features. The abundance of RLE feature vectors compared to the intensity feature vectors enables the proposed algorithm to accurately fine-tune the recognition engine parameters and hence improve the overall system performance.

## Figures and Tables

**Table 1:** Number of transitions/column for all line images in the corpus.

Number of transitions/column	Number of columns	Accumulative percentage
0	3003663	18.44%
1	95418	19.03%
2	7694625	66.27%
3	74196	66.73%
4	4231776	92.71%
5	45013	92.98%
6	1028765	99.30%
7	7403	99.35%
8	94771	99.93%
9	900	99.93%
≥10	10910	100.00%

**Table 2:** Recognition results: monomodels versus trimodels.

Test dataset	Monomodels	Trimodels
Part of training dataset	89.86%	98.78%
Independent of training dataset	89.49%	95.21%

**Table 3:** Recognition results: the proposed algorithm versus Khorsheed [7].

Recognition system	Number of states/model	
	7	16
The proposed algorithm	81.35%	80.44%
Khorsheed [7]	74.58%	5.35%

**Table 4:** Recognition results for each font in the corpus.

Font type	The proposed algorithm	Khorsheed [7]
Simplified	97.26%	88.62%
Thuluth	89.69%	87.85%
Naskh	97.15%	86.45%
Traditional	97.38%	90.05%
Tahoma	99.14%	92.56%
Andalus	98.28%	92.76%
